# Understanding resilience

**DOI:** 10.3389/fnbeh.2013.00010

**Published:** 2013-02-15

**Authors:** Gang Wu, Adriana Feder, Hagit Cohen, Joanna J. Kim, Solara Calderon, Dennis S. Charney, Aleksander A. Mathé

**Affiliations:** ^1^Department of Psychiatry, Icahn School of Medicine at Mount SinaiNY, USA; ^2^Ben-Gurion University of the NegevBeer-Sheva, Israel; ^3^Department of Clinical Neuroscience, Karolinska InstitutetStockholm, Sweden

**Keywords:** resilience, stress, neurobiology, depression, PTSD

## Abstract

Resilience is the ability to adapt successfully in the face of stress and adversity. Stressful life events, trauma, and chronic adversity can have a substantial impact on brain function and structure, and can result in the development of posttraumatic stress disorder (PTSD), depression and other psychiatric disorders. However, most individuals do not develop such illnesses after experiencing stressful life events, and are thus thought to be resilient. Resilience as successful adaptation relies on effective responses to environmental challenges and ultimate resistance to the deleterious effects of stress, therefore a greater understanding of the factors that promote such effects is of great relevance. This review focuses on recent findings regarding genetic, epigenetic, developmental, psychosocial, and neurochemical factors that are considered essential contributors to the development of resilience. Neural circuits and pathways involved in mediating resilience are also discussed. The growing understanding of resilience factors will hopefully lead to the development of new pharmacological and psychological interventions for enhancing resilience and mitigating the untoward consequences.

## Introduction

Resilience is the capacity and dynamic process of adaptively overcoming stress and adversity while maintaining normal psychological and physical functioning (Russo et al., 2012; Rutter, 2012b; Southwick and Charney, 2012). Every individual experiences stressful events and the majority are exposed to trauma at some point during life. Therefore, understanding how one can develop and enhance resilience is of great relevance to not only promoting coping mechanisms but also mitigating maladaptive coping and stress response in psychiatric illnesses such as depression and posttraumatic stress disorder (PTSD). Although the understanding of resilience is overall still at an early stage, recent investigations have identified mechanisms encompassing genetic, epigenetic, developmental, psychological, and neurochemical factors that underlie the development and enhancement of resilience and factors that predict vulnerability to stress and susceptibility to psychiatric disorders in the face of stress and trauma. This review outlines discoveries from recent years from studies that have considerably advanced our understanding of resilience to stress and trauma and will likely move forward the development of pharmacological and psychological interventions for enhancing resilience.

## Genetic factors in resilience

Genetic factors contribute significantly to resilient responses to trauma and stress. A range of human genes and polymorphisms associated with NPY, HPA axis, noradrenergic, dopaminergic and serotonergic systems, and BDNF have been linked to resilient phenotypes (Table 1) (Feder et al., 2009; Russo et al., 2012).

**Table 1:** **Genetic factors in resilience**.

**CNS systems**	**Genes related to resilience**	**Influences of polymorphisms on resilience**	**References**
NPYergic	Neuropeptide Y gene (*NPY*)	Increased susceptibility to anxiety disorders after childhood adversity.	Donner et al., 2012
HPA Axis	CRH receptor 1 gene (*CRHR1*)	Affected the likelihood of developing adult depressive symptoms from child abuse.	Bradley et al., 2008
	FK506-binding protein 5 gene (*FKBP5*)	Predicted severity of adult PTSD symptoms and onset of depression in individuals with childhood trauma.	Binder et al., 2008; Zimmermann et al., 2011
Noradrenergic and Dopaminergic	Catechol-O-Methyltransferase gene (*COMT*)	Influenced the risks of developing PTSD and deficits in stress response and emotional resilience.	Heinz and Smolka, 2006; Skelton et al., 2012
Dopaminergic	Dopamine transporter gene (*DAT1*)	Contributed to susceptibility to PTSD with a history of trauma.	Segman et al., 2002
	Dopamine receptor genes (e.g., *DRD2*, *DRD4*)	Induced differential emotional processing and variability in brain responses to emotional stimuli; Influenced vulnerability to stress and trauma and risk of developing PTSD.	Blasi et al., 2009; Ptacek et al., 2011
Serotonergic	Promoter region of serotonin transporter gene (*5-HTTLPR*)	Short allele strongly associated with increased stress sensitivity and risk for depression upon stress exposure, especially early life stress.	Karg et al., 2011
	Serotonin receptor genes (e.g., *HTR1A*, *HTR3A*, *HTR2C*)	Interacted with environment to mediate stress response and to predict susceptibility to depression.	Gatt et al., 2010; Kim et al., 2011a; Brummett et al., 2012
BDNF	Brain-derived neurotrophic factor gene (*BDNF*)	Interacted with early life stress to predict syndromal depression and anxiety; no clear evidence of association between the Val^66^Met polymorphism and anxiety disorders.	Frustaci et al., 2008; Gatt et al., 2009

### Neuropeptide Y (NPY)

NPY is a neuropeptide that produces anxiolytic effects and promotes protective responses in the face of stress (Wu et al., 2011). Several studies in humans showed that genetic variations of *NPY* contribute to individual susceptibility to stress. One recent study found that two *NPY* haplotypes represented by three single nucleotide polymorphisms (SNPs) correlated with increased susceptibility to anxiety disorders after childhood adversity, and suggested that such behavioral effects can be mediated by altered NPY expression and subsequently dampened HPA-axis responsiveness under the influence of the genetic variation (Donner et al., 2012). Other studies also demonstrated that NPY release was substantially mediated by genetic variations in the *NPY* locus, especially in the promoter region, and that lower haplotype-driven NPY expression predicted weakened resilient response to stress (Zhou et al., 2008; Zhang et al., 2012).

### HPA axis (hypothalamic-pituitary-adrenal axis)

Alterations in genes that regulate HPA-axis functions play an important role in shaping resilience. Polymorphisms in two key HPA-axis genes, *CRHR1* [corticotropin-releasing hormone (CRH) receptor 1 gene] and *FKBP5* (FK506-binding protein 5 gene), have been found to interact with early life stress to predict susceptibility to psychiatric illnesses in adults (Gillespie et al., 2009). One study identified, in two independent populations, significant gene × environment interactions with several individual SNPs of the *CRHR1* gene that influenced the risk of developing adult depressive symptoms in individuals with a history of child abuse (Bradley et al., 2008). The *FKBP5* gene, which is involved in the modulation of glucocorticoid receptor (GR) activity and thereby glucocorticoid signaling, was also found to interact with child abuse through its four SNPs to predict severity of adult PTSD symptoms (Binder et al., 2008). A more recent study showed that interactions between genetic variants of *FKBP5* and early life trauma strongly predicted the onset of depression later in life (Zimmermann et al., 2011).

### Noradrenergic and dopaminergic systems

Polymorphisms in the noradrenergic and dopaminergic systems have also been associated with vulnerability to depression and PTSD. Catechol-O-Methyltransferase (COMT) is an enzyme that metabolizes catecholamines including norepinephrine, epinephrine and dopamine. The *COMT* Val^158^Met polymorphism has been linked to deficits in stress response and emotional resilience, and was found to influence the risk for development of PTSD (Heinz and Smolka, 2006; Skelton et al., 2012). In an important study, Kolassa and colleagues showed that, predictably, higher numbers of different lifetime traumatic event types led to a higher prevalence of lifetime PTSD but that this effect was, in a typical gene-environment interaction fashion, modified by gene polymorphism (Kolassa et al., 2010). Compared to Val^158^Met polymorphism, the low-activity Met/Met homozygotes, with higher levels of norepinephrine and dopamine, exhibited a higher risk for PTSD. Children carrying the Met allele showed a higher cortisol response to stress. However, children who had more stressful life events showed a smaller increase in cortisol, implying that they might be more resilient (Armbruster et al., 2012). This study demonstrated differential effects of genetic and environmental factors on reaction to stress. Polymorphisms in the dopamine receptor genes, including *DRD2* and *DRD4*, and in the dopamine transporter gene *DAT1*, have also been implicated in stress responsivity, emotion processing, and susceptibility to PTSD and depression (Segman et al., 2002; Dunlop and Nemeroff, 2007; Blasi et al., 2009; Ptacek et al., 2011; Skelton et al., 2012).

### Serotonergic system

Studies of polymorphic traits of the serotonin transporter gene *SLC6A4* and receptor genes have led to several discoveries regarding the effects of gene × environment interactions on resilience. A recent meta-analysis of 54 human studies confirmed that the interaction of stress exposure and the polymorphism in the promoter region of the serotonin transporter gene (*5-HTTLPR*) is strongly associated with stress sensitivity and risk for depression, with the short, less transcriptionally efficient s-allele being linked to increased stress sensitivity and risk of developing depression upon stress exposure (Karg et al., 2011). A particularly strong association between the s-allele and risk of developing depression was found in the group with a history of childhood maltreatment (Karg et al., 2011; Southwick and Charney, 2012). The s-allele of the *5-HTTLPR* gene was also found, in two independent populations, to interact with childhood and adult traumatic experiences to increase the risk for PTSD (Xie et al., 2009). Polymorphisms in several serotonin receptor genes, such as *HTR1A*, *HTR3A*, and *HTR2C*, have been shown to interact with stressful life environment as well as polymorphisms from other genes (e.g., Val^66^Met in the *BDNF* gene) to predict susceptibility to depression (Kim et al., 2007, 2011a; Gatt et al., 2010), and to mediate HPA-axis activation and emotional response to stress (Brummett et al., 2012).

### Brain-derived neurotrophic factor (BDNF)

The role of the *BDNF* Val^66^Met polymorphism in stress response and resilience has not been clarified. A meta-analysis of seven studies found no significant association between the Val^66^Met polymorphism and anxiety disorders (Frustaci et al., 2008). Specifically, two case-control studies of PTSD found no significant association between the Val^66^Met polymorphism and PTSD diagnosis (Rakofsky et al., 2012). One study, however, showed that the Val^66^Met polymorphism interacted with early life stress to predict syndromal depression and anxiety, with higher depression in Met carriers (Met/Met and Met/Val) and higher anxiety in Val/Val genotype, indicating that both alleles, interacting with exposure to early life stress, may contribute to mechanisms of distinct risks (Gatt et al., 2009).

The field of genetics is now moving rapidly to genome-wide studies on large populations to examine the complex genetic contributions to resilience, with additional genetic polymorphisms, gene-by-gene and gene-by-environment interactions being currently identified. As the genetic underpinnings of resilience become better illuminated, it is anticipated that gene and drug therapies can be developed specifically for genetic profiles of low resilience.

## Epigenetic factors in resilience

Epigenetics refers to functional modifications to the genome without change in the DNA sequence. Such modifications serve to regulate gene expression and phenotype through mechanisms such as DNA methylation and demethylation, as well as histone modifications including methylation, acetylation, and phosphorylation. Epigenetic differences can be a consequence of exposure to stress-related factors during critical periods of development, and hence contribute to susceptibility to psychiatric disorders (Tsankova et al., 2007; Dudley et al., 2011).

Several animal studies have found that histone acetylation or phosphoacetylation in several subregions of the hippocampus increased after exposure to acute stressors (social defeat stress, forced swim stress, and predator stress) in both mice and rats, suggesting an adaptive role of these epigenetic changes in memory formation and stress response (McGowan et al., 2011; Sun et al., 2013). Intracerebral or systemic administration of histone deacetylase inhibitors (HDACi), alone or combined with antidepressants, resulted in antidepressant-like responses in several animal models (Sun et al., 2013). Histone methyltransferases (e.g., GLP, SUV39H1, G9a) are down-regulated in the nucleus accumbens of susceptible mice exposed to chronic social defeat stress, while these molecules were up-regulated in resilient mice exhibiting low depression-like responses, suggesting that histone methylation may be adaptive in the face of stress and protect against development of depression (Covington et al., 2011). Maternal care was found to influence stress response through epigenetic alterations, with offspring of high maternal care showing increased hippocampal GR expression and enhanced glucocorticoid negative feedback sensitivity, and hence more modest HPA response to stress, through hypomethylation at the NGFI-A nerve growth factor-inducible protein A (NGFI-A) binding site of a GR promoter (Weaver et al., 2004).

Human studies have begun to identify the effects of epigenetic changes on the regulation of the stress response. Suicide victims with childhood abuse had increased methylation of a GR (*NR3C1*) promoter in the hippocampus, and thereby decreased hippocampal GR expression, compared to suicide victims without childhood abuse and to control subjects (victims of sudden, accidental death without childhood abuse) (McGowan et al., 2009). This finding is consistent with those from animal studies showing that history of early adversity is associated with GR expression and stress response in adulthood. Another study showed that DNA methyltransferase (DNMT) expression was altered in a region-specific manner in the brains of suicide victims compared to controls who died of causes other than suicide (Poulter et al., 2008). This study found increased DNMT-3B expression in the prefrontal cortex (PFC), and an associated increase in DNA methylation of the promoter region of the γ-aminobutyric acid (GABA) A receptor subunit alpha-1 gene (*GABRA1*), the product of which was previously demonstrated to be down-regulated in the brains of suicide victims (Merali et al., 2004). Higher methylation of *MAN2C1*, a gene that encodes α-mannosidase, was shown to interact with greater exposure to potentially traumatic events to predict an increased risk of lifetime PTSD (Uddin et al., 2011). A number of epigenetic studies in animal models and humans investigating the association between epigenetic changes and risk for maladaptive stress responses and mental illnesses have recently been published (Radley et al., 2011; Schmidt et al., 2011; Murgatroyd and Spengler, 2012; Rusiecki et al., 2012).

## Developmental factors in resilience

Developmental environment is another crucial contributor to resilience (Rende, 2012). Severe adverse events in childhood can negatively affect the development of stress response systems, in some cases causing long-lasting damage. Numerous rodent and primate studies suggest that animals abused by their mothers in the first few weeks of life show both delayed independence and decreased stress management skills in adulthood (Feder et al., 2011). These changes are reflected in abnormally high anxiety levels, increased HPA axis activity, and increased basal CRH levels in the cerebrospinal fluid (CSF) (Strome et al., 2002; Claes, 2004; McCormack et al., 2006). It is important to note that non-human primates, who have suffered childhood abuse, resulting in damaged stress response systems, may be more likely to abuse their own children (Maestripieri et al., 2007). In this way, the cycle of abuse is continued through generations.

Similar long-lasting alterations, including changes in the central nervous system (CNS) circuits, have been found in studies of human survivors of childhood trauma (Heim et al., 2010). Prenatal stress and childhood trauma have been linked to a hyperactive HPA axis with attendant risk of negative effects of chronic hypercortisolemia later in life (Frodl and O'Keane, 2012). Furthermore, severe early life stress leads to hyperfunctioning of the locus coeruleus-norepinephrine (LC-NE) system in adulthood (Feder et al., 2011). One study of police recruits with a history of childhood trauma found that in contrast to controls, the police subjects had significantly higher levels of a salivary metabolite of norepinephrine when watching aversive videos (Otte et al., 2005). Childhood abuse can lead to a reduction of hippocampal volume, which is frequently seen in patients with mood disorders (Janssen et al., 2007; Davidson and McEwen, 2012). As the hippocampus is one of the most plastic regions of the brain, there is hope that pharmacological treatments, such as antidepressants, may be able to reverse this decrease in volume by increasing neural progenitor cells (Boldrini et al., 2012). PET studies have also revealed decreased activation in the hippocampus during memory tests in patients with a history of childhood abuse (Heim et al., 2010). Other brain areas seem to be affected by childhood abuse as well. For instance, a recent study suggests that childhood maltreatment has a pronounced effect on two separate neuroimaging markers—reduced hippocampal volume and amygdala responsiveness to negative facial expressions (Dannlowski et al., 2012). Chronic, unmanageable social and psychological stress, and maltreatment, especially early in life, are also linked to shorter telomeres, which have been associated with increased risk of developing somatic diseases such as cancer, diabetes and heart diseases, and psychiatric disorders, particularly depression (Blackburn and Epel, 2012; Price et al., 2013).

Certain factors play major roles in determining whether a childhood traumatic event will lead to vulnerability or instead, to resilience. One of these factors is the degree of control that the person has over the stressor (Feder et al., 2011). Episodes of early uncontrollable stress can lead to “learned helplessness,” where a person is conditioned to believe that they are unable to change the circumstances of their situation (Overmier and Seligman, 1967). Learned helplessness is also used as a model for depression in animals. When administered inescapable and erratic shocks, animals tend to develop heightened anxiety states and fear responses (Overmier and Seligman, 1967). Furthermore, their active coping is reduced when faced with later stressors. Learned helplessness in animals is also believed to lead to dysregulation of serotonergic neurons in the dorsal raphe nuclei (Greenwood et al., 2003), as well as a reduction of cell proliferation in the hippocampus (Ho and Wang, 2010). These dysregulations are likely to have severe negative repercussions on both cognition and mood.

On the other hand, when animals are administered shocks that are avoidable by behavioral modification, learned helplessness does not seem to develop (Seligman and Maier, 1967). In this same way, humans that have been able to successfully master a mild or moderate stressor (for example, the end of a friendship or illness of a parent) appear to be resilient to a variety of other later stressors (Feder et al., 2009; Russo et al., 2012). This phenomenon is called “stress inoculation,” and occurs when the person develops an adaptive stress response and a higher-than-average resilience to negative effects of subsequent, uncontrollable stressors (Southwick and Charney, 2012). Stress inoculation is a form of immunity against later stressors, much in the same way that vaccines induce immunity against disease (Rutter, 1993). Research in rodents supports the stress inoculation hypothesis and has suggested that this protection against some of the later negative effects may be due to neuroplasticity in the PFC induced by stress inoculation (Southwick and Charney, 2012). In one study, young monkeys were presented with a controllable stressor (periodic short maternal separations) over a course of 10 weeks (Parker et al., 2004). These monkeys experienced acute stress during the separation periods, illustrated by agitation as well as temporary increased levels of cortisol. Yet, at 9 months of age, they experienced less anxiety and lower basal stress hormone levels than monkeys who did not undergo the separations. Additionally, at later time points, the group of stress-inoculated monkeys showed higher cognitive control, higher curiosity in a stress-free situation and larger ventromedial PFC volume (Parker et al., 2005; Lyons et al., 2009).

It is important to note that although research has outlined numerous ways in which developmental environment can negatively impact a person, resilience is in fact a common trait, following even the most severe adversities. Between 50 and 60% of the general population experience a severe trauma during their lifetime, yet the prevalence of PTSD is estimated at 7.8% (Russo et al., 2012). Other studies have found that neural circuits involved in resilience can be modified for many years after adversity. For instance, the majority of adolescents whose development was stunted in childhood due to trauma were able to developmentally “catch-up” when relocated to a supportive, loving environment (Masten, 2001; Rutter, 2012a). The fact that not all animals or humans exposed to uncontrollable traumatic experiences develop stress-related disorders clearly implies that environmental factors interact with genetic endowment and together, affect resilience. In fact, resilient genes may be sufficient to help a person overcome the most traumatic developmental events in some cases (Feder et al., 2011).

### Implications for promoting resilience in child rearing

The findings that the developmental environment has significant effects on building and enhancing resilience from a young age impart clear messages for child rearing. Several large-scale longitudinal studies have investigated resilience in participants from childhood or adolescence through the transition to adulthood. Results from these studies strongly indicated that key factors including positive family functioning and peer relationships, connections to supportive adults and prosocial romantic partners, planfulness, self-discipline, and cognitive ability, all contribute to a more successful transition to adulthood and more resilient functioning (Burt and Paysnick, 2012). Interventional paradigms in the form of foster care, adoption, and parent training can improve the quality of parenting, family function, and attachment relationship, and in turn promote adaptive functioning and resilience in children and youth (Sapienza and Masten, 2011).

Children with a history of maltreatment showed lower resilient functioning than those without maltreatment (Cicchetti and Rogosch, 2012). Children with exposure to war and related traumatic experiences (e.g., child soldiers, rape, bombing, forced displacement) showed increased risks for PTSD as well as other medical conditions such as cardiovascular diseases in adulthood (Werner, 2012). Protective factors against deleterious impact of war-related adversities in children include a strong, positive bond between the primary caregiver and the child, the social support from teachers and peers, a shared sense of values, religious beliefs that find meaning in suffering, and humor and altruism as defense mechanisms (Werner, 2012). Besides children from an abusive and life-threatening environment, a newly identified group at risk is youth from affluent families, who may face higher risk of adjustment problems (e.g., substance use, depression, and anxiety) (Luthar and Barkin, 2012). Parents' lax repercussions on discovering substance use was shown to be a major vulnerability factor. Moreover, the levels of teens' symptoms (rule breaking, anxious-depressed, and somatic symptoms) were found to correlate more strongly with the teens' relationships with mothers than with fathers, which may in part reflect greater amount of time spent with mothers, who are generally the primary caregivers of their children. Therefore, positive changes in parenting for affluent youth are of critical importance, including adopting a strict zero-tolerance policy regarding students' law breaking, remaining vigilant about their children's activities outside school, and engaging in talks and workshops for families in distress and holding support groups particularly for mothers (Luthar and Barkin, 2012).

A review of efficacy of different interventions for children and adolescents with a history of trauma exposure indicates that cognitive-behavioral treatment, in both individual and group formats, is effective in reducing psychological harm such as anxiety and depressive disorders and symptoms (Wethington et al., 2008). Stress inoculation training (SIT), a preventive and interventional cognitive-behavioral paradigm, has been shown to be helpful in reducing anxiety and stress-related symptoms in adolescents (Maag and Kotlash, 1994). School-based interventions, including SIT, can improve adaptive coping skills and decrease the likelihood of developing PTSD symptoms in children exposed to war (Werner, 2012).

**Figure 1:**
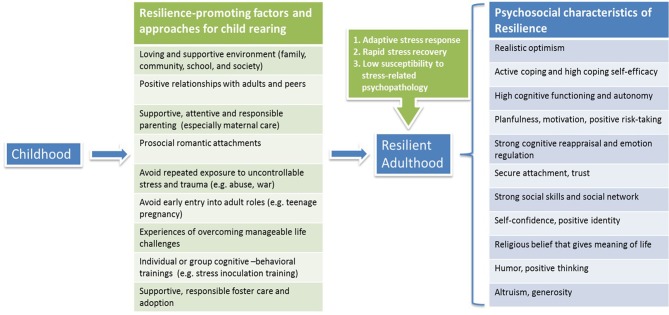
**Promoting resilience in child rearing**.

In summary, it is critical to provide children with a loving, healthy and supportive environment as they grow up, to avoid exposing them to repeated unmanageable stress, and to offer them chances to embrace and conquer life challenges so as to develop mastery of critical life stressors and acquire “stress inoculation” (Southwick and Charney, 2012). Education on successful parenting should be able to help to foster children in a resilience-promoting environment and to minimize occurrence of impaired stress response through generations. Moreover, training programs for children that focus on constructing and maintaining supportive social networks, enhancing prosocial behavior and cognitive reappraisal, and promoting coping self-efficacy and self-esteem, can all contribute to resilience building from an early age (Figure 1).

## Psychological factors in resilience

Significant research has been done on the psychosocial factors of stress tolerance and resilience building (Duryea et al., 1990; Chemtob et al., 1997; Pietrzak et al., 2010). Cognitive processes, personality traits, and active coping mechanisms, among others, contribute to resilience. These qualities also interact with biological factors to enhance adaptation in the face and aftermath of traumatic events, and confer resilience (Charney, 2004).

### Individual characteristics and behaviors

Characteristics such as high level of intellectual functioning, efficient self-regulation, active coping styles, optimism, and secure attachment were observed in youth who had faced adverse situations and settings, yet did not succumb to the adverse impact of extreme stress (Richardson, 2002).

#### Optimism

Positive affect has been found to be protective in the face of stress in numerous studies. In addition to decreasing autonomic arousal upon stress exposure (Folkman and Moskowitz, 2000), positive affect is also associated with quicker recovery times and better overall physical health (Scheier et al., 1989; Warner et al., 2012). Similarly, optimism, herein defined as the expectation for good outcomes, has been consistently associated with the employment of active coping strategies, subjective well-being, physical health, and larger and more fulfilling social networks and connections (Stewart and Yuen, 2011; Galatzer-Levy and Bonanno, 2012; Gonzalez-Herero and Garcia-Martin, 2012; Colby and Shifren, 2013). Unlike pessimists, optimists reported less hopelessness and helplessness and are less likely to use avoidance as a coping mechanism when under duress (e.g., among breast cancer patients) (Carver et al., 2010).

#### Cognitive reappraisal

Strongly associated with resilience is the ability to monitor and assess negative thoughts and replace them with more positive ones, or cognitive reappraisal (McRae et al., 2012). Known as cognitive flexibility or cognitive reframing, this emotion regulation strategy involves changing the way one views events or situations. Consciously reassessing adverse or traumatic events to find the silver lining is associated with resilience (Gross, 2002). Viktor Frankl, the author of *Man's Search for Meaning* and the founder of logotherapy, attributed his psychological endurance and survival of concentration camps mainly to “meaning finding,” the belief that the striving to find a meaning in one's life is the most important, powerful motivating and driving force to continue living (Frankl, 2006). In a study examining cognitive protective factors in the face of stress, women with high cognitive reappraisal ability exhibited less depressive symptoms than their cohorts with low cognitive reappraisal ability (Troy et al., 2010). Attachment style may also play a role in reappraisal ability and resilience. In a study of 632 men and women, researchers found that secure attachment was associated with higher cognitive reappraisal and resilience and that these two factors partially mediated individuals' well-being (Karreman and Vingerhoets, 2012). Securely attached participants were more likely to reframe situations as less emotional and less likely to suppress emotional expression. As expected, preoccupied attachment was inversely related to well-being due to less utilization of cognitive reappraisal.

A possible gender difference in emotional regulation/cognitive reappraisal is of note. Neural data suggest that women might employ positive emotions to help them regulate their emotions to a larger extent than men; it is possible that in men, use of emotion regulation is more automatic (McRae et al., 2008). Utilizing a randomized control design, an intervention study in Israeli citizens under ongoing war stress found that gender might act as a moderator in the development of resilience and reduction of helplessness (Farchi and Gidron, 2010). While the “psychological inoculation” intervention was expected to increase coping self-efficacy and to improve mental resilience more so than ventilation, the intervention's efficacy differed by sex. Psychological inoculation, possibly augmenting self-efficacy and hope, appeared to decrease helplessness in men, while the ventilation intervention appeared to decrease helplessness in women. The ventilation intervention may have had calming effects and lent a sense of connectedness that was helpful to women.

#### Active coping

Coping, using behavioral or psychological techniques utilized to reduce or overcome stress, has been linked to resilience in individuals (Feder et al., 2009) and is coming to be recognized for its intervention potential (Taylor and Stanton, 2007). The literature distinguishes between active coping, involving behavioral and/or psychological strategies to change qualities of the stressor, the stressor itself, or how the stressor is perceived, and avoidant coping, involving activities and mental processes that are employed in lieu of dealing directly with the stressful trigger (Chesney et al., 2006). Emotional or behavioral withdrawal, alcohol use, and other substance use are classic examples of avoidant coping behavior (Lawler et al., 2005). While individuals who primarily exercise avoidant coping are at risk of psychological distress and subsequent negative responses, active coping has consistently been associated with adaptability and psychological resilience (Holahan and Moos, 1987; Moos and Schaefer, 1993). Among chronic pain patients, passive coping strategies were correlated with psychological distress and depression, while active coping strategies were inversely correlated with psychological distress (Snow-Turek et al., 1996). In a study examining two groups of Israeli veterans and former POWs, Solomon and colleagues found that high sensation seeking and low sensation seeking POWs significantly differed in their subjective assessments of suffering, use of coping methods, and emotional states while in prison (Solomon et al., 1995). Low sensation seeking former POWs reported more symptoms of PTSD and other psychiatric symptoms. Further distinguishing coping styles, task-oriented coping was positively correlated with resilience while emotion-oriented coping was related to low resilience among undergraduate students (Campbell-Sills et al., 2006). Drawing a relationship with personality, resilience among these young adults was inversely related with neuroticism but positively so with extraversion and conscientiousness. Even among sport performers, individuals with high hardiness or resilience tend to employ active coping strategies during stressful (competitive) situations compared with low hardiness groups (Hanton et al., 2013).

#### Social support

Both the presence of social support and the behavior of seeking social support have been associated with psychological hardiness and flourishing in the face of major adverse life events (Ozbay et al., 2008). The inverse also appears to be true; poorer social support has been linked to psychiatric disorders including PTSD (Tsai et al., 2012). Research with cancer patients found depression to be correlated with poor social support and higher external locus of control (Grassi et al., 1997). Depressed patients consistently reported weak or a lack of support from family, friends, and other social contacts (such as neighbors, colleagues, and less intimate relatives). Such patients were also often characterized by early maladjustment to their diagnosis of cancer (Grassi et al., 1997).

#### Humor

Humor has been identified as a form of active coping contributing to resilience not only for its capability for alleviating tension and but also for its ability to attract social support (Vaillant, 1992). Humor is widely used by veterans, repatriates, terminally ill patients, and youth alike and has been shown to be protective against stress (Southwick and Charney, 2012). Cameron and colleagues employed an ecological research method to examine the type and role of humor in resilient adolescents' daily social functioning and found that humor served various socioemotional functions and was a buffer in risky situations (Cameron et al., 2010). In a study of 215 sojourn students from Mainland China studying at a Hong Kong university, humor was seen as imperative to students' ability to adjust to the new culture and thrive in the face of acculturative stress (Cheung and Yue, 2012). In fact, humor increased with an increase in frequency of acculturative hassles.

#### Physical exercise

Physical exercise has positive effects on psychological well-being as well as mood, clinical depression, and self-esteem. Physical exercise has been shown to affect neurobiological factors of resilience in animal (Fleshner et al., 2011) and human studies (Wittert et al., 1996; Winter et al., 2007). In a 10-year study of 424 depressed adult patients, Harris and colleagues examined the relationship between physical activity, exercise coping and depression at 1-year, 4-year, and 10-year follow-up points (Harris et al., 2006). While no significant relationship between physical activity and subsequent depression was found, physical activity was negatively correlated with concurrent depression. In other words, physical activity may be beneficial to those currently depressed or facing major stressors. Moreover, in a rat model of depression, voluntary running had antidepressant-like effects in behavioral tests and in parallel enhanced NPY expression and neurogenesis (Bjornebekk et al., 2005, 2006).

#### Prosocial behavior

Altruism has also been associated with resilience in both adults and children (Southwick et al., 2005; Leontopoulou, 2010). Staub and Vollhardt examined case studies and qualitative studies where individuals' victimization and suffering bred prosocial behavior, ultimately promoting recovery from trauma, post-traumatic growth, and resilience, and suggested that post-traumatic interventions may promote “altruism born of suffering” (Staub and Vollhardt, 2008). A study of 232 elementary school children in Greece showed that higher altruism resulted in lower classroom competitiveness and was associated with higher empathy and resilience (Leontopoulou, 2010). Studies also show the birth of prosocial behavior and action from trauma enduring during times of civil conflict and unrest as a byproduct of personal healing (Hernández-Wolfe, 2010).

#### Trait mindfulness

Trait mindfulness is another psychological factor associated with resilience. Originated as a Buddhist meditation practice, mindfulness concentrates on moment-to-moment awareness of bodily activities, feelings, emotions, or sensations, while purposely perceiving and discarding any distracting thoughts that come into awareness (Thompson et al., 2011). Studies on trait mindfulness suggest that strong pre-trauma mindfulness skills may help prevent ruminative, depressogenic thinking, thereby counteracting the development of depression and PTSD symptoms following trauma (Thompson et al., 2011). A study of 124 firefighters showed that trait mindfulness was negatively related to depressive and PTSD symptoms, physical symptoms, and alcohol problems, suggesting that trait mindfulness may reduce avoidant coping in response to stress and contribute to resilience (Smith et al., 2011).

### Moral compass

The existence of a moral compass or an internal belief system guiding values and ethics is commonly shared among resilient individuals (Southwick et al., 2005). Though religion or spirituality is often a facet in one's moral compass, the concept of a moral compass is grounded in a more innately human belief in morality. A study of 121 outpatients diagnosed with depression and/or an anxiety disorder showed that a low or lack of purpose in life and less frequent physical exercise were correlated with low resilience, but low spirituality prevailed as a leading predictor of low resilience (Min et al., 2012). Similarly, purpose in life was a key factor linked to resilience in a study of 259 primary care patients with a history of exposure to a range of severe traumatic events (Alim et al., 2008).

## Neurochemical factors in resilience

A number of neurochemicals have been found to be involved in resilience. These neurochemicals have been shown to interact with and to balance each other to produce regulatory effects on acute and long-lasting adaptations to stress.

### NPY

NPY is widely distributed in the brain (Wu et al., 2011; Sah and Geracioti, 2012). It counteracts anxiogenic effects of CRH in several brain regions that regulate stress and anxiety, including the hypothalamus, hippocampus, amygdala, and locus coeruleus (Sajdyk et al., 2004). Many studies on animal models and humans have confirmed the beneficial role of NPY in mediating resilience and vulnerability to stress and anxiety. Animals with PTSD-like behaviors showed a significant down-regulation of NPY in several brain regions including the amygdala and hippocampus, and centrally administered NPY reversed the negative behavioral effects of predator-scent stress (Cohen et al., 2012). Human studies found that, under uncontrollable stress induced by harsh military training, plasma NPY levels were markedly increased, and higher NPY levels were associated with better behavioral performance and stress response (Morgan et al., 2000, 2002). Higher plasma NPY levels were also found in combat-exposed veterans without PTSD than in those with PTSD (Yehuda et al., 2006). Significantly lower NPY levels in CSF were found in men with combat-related PTSD compared to healthy controls without PTSD (Sah et al., 2009). Thus, a wealth of studies indicate a positive correlation between NPY levels and resilience to deleterious effects of stress, and suggest a potential pharmacotherapeutic target for effectively reducing anxiety and enhancing resilience to adversity and stress. Studies are currently being conducted in this regard, using possibly effective delivery routes such as intranasal administration.

### HPA axis

Upon stress exposure, CRH is released from the hypothalamus and acts on the pituitary gland, causing it to release adrenocorticotropic hormone (ACTH), which in turn stimulates the adrenal cortex to release cortisol and dehydroepiandrosterone (DHEA). Cortisol exerts negative feedback effects on the hypothalamus and pituitary, suppressing CRH and ACTH production, while DHEA is thought to have anti-glucocorticoid effects by inhibiting or blocking the effects of cortisol (Jones and Moller, 2011). This complex set of feedback interactions constitutes the HPA axis, which is a key neuroendocrine player modulating behavioral responses to stress (Russo et al., 2012).

Cortisol levels are linked to risk and resilience to stress-related psychiatric disorders, with higher levels associated with depression (Nemeroff and Vale, 2005), and lower levels with PTSD either as a possible trait that predisposes to the development of PTSD or as a consequence of trauma (Radley et al., 2011; Binder and Holsboer, 2012). DHEA together with DHEA sulfate (DHEA-S), have also been implicated in stress response and psychiatric disorders, with lower levels of DHEA(S) associated with depression, and elevated levels of DHEA(S) associated with PTSD (Maninger et al., 2009; Rasmusson et al., 2010). Of note, some studies have generated mixed findings (Hoge et al., 2007; Maninger et al., 2009). Because cortisol and DHEA(S) are released synchronously and function together through their antagonistic, dualistic homeostasis, the DHEA(S)/cortisol ratio has been found to be a crucial parameter that indicates differential stress vulnerability (Morgan et al., 2004; Markopoulou et al., 2009; Jones and Moller, 2011; ó Hartaigh et al., 2012).

CRH and its two receptors, CRHR-1 and CRHR-2, are important mediators of stress response (Southwick et al., 2005). In depression and PTSD, increased CRH levels in CSF have been found, which may relate to the dysregulation of signal transduction via the two receptors (Charney, 2004). CRHR-1 and CRHR-2 are differentially distributed in the brain, with CRHR-1 primarily found in the neocortex, basolateral amygdala, and hippocampus, and CRHR-2 in the lateral septum, medial and cortical nuclei of the amygdala, and dorsal raphe (Holsboer and Ising, 2010). CRHR-1 signaling plays a crucial role in anxiogenic circuits and contributes to anxiety-like response to stress. Consequently, preclinical and clinical studies have examined the antagonism of CRHR1 as a potential therapeutic intervention targeting aberrant CRH levels in mood and anxiety disorders and have generated some encouraging results (Paez-Pereda et al., 2011). CRHR-2 mainly modulates the effects of CRHR-1 signaling and can be either anxiolytic or anxiogenic depending on the circumstances (Hauger et al., 2009; Binder and Nemeroff, 2010).

### Noradrenergic and dopaminergic systems

The noradrenergic system is activated upon stress, resulting in increased release of norepinephrine primarily from the locus coeruleus to its many projection sites that modulate stress responses and emotional behaviors, including the amygdala, hippocampus, hypothalamus and PFC, all of which constitute the LC-NE system (Aston-Jones and Cohen, 2005; Strawn and Geracioti, 2008). The activation of the LC-NE system under acute stress leads to generation and transmission of negative emotional memories starting from the amygdala, a process that can be inhibited by blocking norepinephrine activity (Charney, 2004). Hyperresponsiveness of the LC-NE system may result in chronic anxiety and fear (Feder et al., 2009). An imaging study in humans showed that disinhibited norepinephrine signaling may contribute to the etiology of PTSD by enhancing basolateral amygdala responses to fear stimuli (Onur et al., 2009). The norepinephrine transporter (NET) and receptors (α- and β-adrenoreceptors) involved in norepinephrine signaling have been implicated as biological mediators of stress-related psychiatric disorders and resilience (Krystal and Neumeister, 2009; Jhaveri et al., 2010). Dopamine release upon stress is increased in the PFC and inhibited in the nucleus accumbens, an area mainly associated with the reward pathway (Charney, 2004). Some studies have found decreased levels of circulating dopamine in depression and elevated urinary and plasma dopamine concentrations in PTSD (Charney, 2004; Dunlop and Nemeroff, 2007). A recent imaging study in humans showed that striatal dopamine transporter (DAT) density was higher in PTSD patients than in traumatized controls, suggesting a possible higher dopamine turnover in PTSD that can contribute to potentiation of exaggerated fear response to a stressful stimulus (Hoexter et al., 2012). Dopamine D_1_ and D_2_ receptors can form heterodimers by binding directly to each other, and these heterodimers were markedly elevated in the striatum in postmortem brains from patients with depression (Pei et al., 2010). Disrupting the coupling of D_1_ and D_2_ receptors has been shown to produce antidepressant-like effects, providing a possible novel target for antidepressant treatment (Pei et al., 2010; Wong and Liu, 2012).

### Serotonergic system

Serotonin is one of the most studied neurotransmitters in relevance to mood and anxiety. Acute stress leads to increased serotonin turnover in multiple brain areas, including the amygdala, hypothalamus, PFC and nucleus accumbens (Feder et al., 2009). Serotonin affects the regulation of stress response and emotional behaviors through 5-HT_1−7_ receptors in separate brain regions. The 5-HT_1A_ receptor is anxiolytic and may play an important role in the etiology of anxiety disorders. Animal studies have found anxiety-like behaviors after knocking out 5-HT_1A_ (Akimova et al., 2009). A few human imaging studies have also showed decreased 5-HT_1A_ binding and functioning in the amygdala, anterior cingulate cortex and raphe nuclei in patients with anxiety disorders compared to healthy controls (Akimova et al., 2009). The 5-HT_2A_ receptor, on the other hand, is thought to be anxiogenic, and 5-HT_2A_ antagonists prevent anxious behavior and dysregulated stress responses following early life stress (Benekareddy et al., 2011). Other serotonin receptors (such as 5-HT_1B_ and 5-HT_2C_) have also been implicated in adaptive responses to stress (Krystal and Neumeister, 2009). For example, overexpressing 5-HT_1B_ in the caudal dorsal raphe nucleus led to reduced conditioned fear and helplessness in animal stress models (McDevitt et al., 2011).

### BDNF

BDNF, a neurotrophic factor expressed in various brain regions including the amygdala, hippocampus, PFC and basal forebrain, is implicated in mood and anxiety disorders (Yamada and Nabeshima, 2003; Angelucci et al., 2005; Duman, 2009). BDNF supports neuronal proliferation, differentiation and growth during development, and promotes neuronal survival and functioning in adulthood (McAllister, 2002). Several studies have shown down-regulation of BDNF in the hippocampus after exposure of animals to various types of stress, and in postmortem studies of suicide-depression patients (Duman and Monteggia, 2006; Duman, 2009). Hippocampal BDNF expression contributed critically to resilient adaptations to chronic stress (Taliaz et al., 2011). BDNF acts through its two main receptors, TrkB and p75 (Castren and Rantamaki, 2010). The BDNF-TrkB pathway has been associated with both PTSD in humans and in animal models of fear conditioning, extinction and inhibitory learning (Mahan and Ressler, 2012). Central administration of BDNF has antidepressant-like effects and can enhance hippocampal neurogenesis (Li et al., 2008; Autry and Monteggia, 2012). Evidence from animal and human studies shows that administration of antidepressants can lead to increase of BDNF and TrkB expression in the hippocampus and PFC, suggesting a role of BDNF-TrkB signaling in the behavioral effects of antidepressants (Masi and Brovedani, 2011). Nevertheless, there is also evidence for antidepressant effects without changes in BDNF or neurogenesis (David et al., 2009; Petersen et al., 2009; Hansson et al., 2011). Much less work has been done regarding the exact role of the BDNF-p75 signaling pathway in resilience, probably due to the low affinity of p75 (Numakawa et al., 2010).

### Glutamate, GABA, and endocannabinoids

Glutamate, GABA, and endocannabinoids have also been widely studied and implicated in the stress response, resilience, and pathophysiology of mood and anxiety disorders (Harvey and Shahid, 2012; Hill, 2012; Sanacora et al., 2012). The dysregulation of these systems can lead to profound deficits in successful adaptation to acute and chronic stress. Pharmacological studies targeting these systems in psychiatric disorders have begun to show promising results in achieving therapeutic effects (Hill and Gorzalka, 2009; Murrough and Charney, 2010; Kirilly et al., 2012; Mathew et al., 2012; Mathews et al., 2012).

## Neural circuitry of resilience

Animal and human studies have investigated the brain circuits implicated in mood and anxiety and have shown that dysregulated functions and interactions among these circuits can result in low resilience phenotypes (Feder et al., 2009; Franklin et al., 2012). The reward and fear circuits play critical roles in the development of resilient character traits and adaptive social responses to stress.

### Neural circuitry of reward

Enhanced functioning of the reward circuitry contributes to resilience to stress and trauma (Charney, 2004). A key reward circuit is the mesolimbic dopamine pathway, which carries dopamine signaling from the ventral tegmental area of the midbrain to the nucleus accumbens in the limbic system, and also to other brain regions such as the amygdala, hippocampus, and medial PFC. The mesolimbic dopamine pathway is linked to behavioral responses to rewards (e.g., food, sex, and drugs of abuse), and functional abnormalities in this pathway can contribute notably to key depressive symptomatology such as anhedonia, decreased energy, and reduced motivation seen in individuals with depression (Nestler and Carlezon, 2006). Studies have shown that the onset of depression is likely to happen during adolescence, when reward functioning is generally higher than during childhood and adulthood, and that increased reactivity in the medial PFC and decreased reactivity in the striatum are implicated in adolescent depression (Forbes and Dahl, 2012). Children of depressed parents, therefore at high risk for depression, showed altered amygdala and nucleus accumbens activation to affective stimuli compared to those of non-depressed parents, therefore at low risk for depression (Monk et al., 2008). Depressed and PTSD patients showed weakened responses to rewards in the striatal areas including the nucleus accumbens (Sailer et al., 2008; Pizzagalli et al., 2009). Deep brain stimulation in the nucleus accumbens has antidepressant, anti-anhedonic and anxiolytic effects in patients with treatment-resistant depression, suggesting that modulating a dysfunctional reward system can lead to improvement of the core symptoms in depression (Schlaepfer et al., 2008; Bewernick et al., 2010). Although compelling evidence has shown that an enhanced, highly functional reward system may be beneficial for positive, adaptive response to stress, one study found that Special Forces soldiers of high resilience showed less activation in the subgenual PFC and nucleus accumbens under a high-reward condition compared to healthy civilian controls, suggesting that a potentially “sturdy” reward system may contribute to resilience (Vythilingam et al., 2009). The exact role of the reward system and the associated neurotransmitters in the development of resilience and pathophysiology and even etiology of stress-related psychiatric disorders needs further elucidation.

### Neural circuitry of fear

Resilience to extreme stress entails the ability to avoid excessive overgeneralized fear responses and to enhance favorable reconsolidation and extinction processes related to fear memories (Charney, 2004). Several studies have identified the components of the neural circuitry of fear response, which includes the amygdala, hippocampus, medial PFC, nucleus accumbens, ventromedial hypothalamus, and a number of brain stem nuclei (Davis, 2006; Maren, 2008; Quirk and Mueller, 2008). These regions play key roles in fear processing including the fear learning/conditioning, perception of threat, execution of efferent components of fear response, and modulation of fear memories through potentiation, consolidation, reconsolidation, and extinction (Shin and Liberzon, 2010). Patients with PTSD showed hyperactivation in the amygdala and hypoactivation in the ventromedial PFC and anterior hippocampus, which may indicate reduced top-down inhibition of the amygdala and account for exaggerated fear responses (Etkin and Wager, 2007). Other brain regions such as the dorsal anterior cingulate cortex and insular cortex have also been implicated in the maladaptive regulation of fear responses in PTSD, with some studies showing hyperresponsiveness and some showing hyporesponsiveness of these regions (Shin and Liberzon, 2010). Compared to trauma victims without PTSD, individuals with PTSD demonstrated behavioral sensitization to stress, overgeneralization of the conditioned stimulus (CS)-unconditioned stimulus (US) response, impaired CS-US pairings and impaired fear inhibitory learning, all of which are thought to be characteristic of dysregulated fear responses and can result in the core symptoms seen in PTSD, such as intrusive memories and flashbacks, enhanced avoidance of reminders, and autonomic hyperarousal (Mahan and Ressler, 2012). One study found higher potentiation of the startle response to safety cues in patients with PTSD compared to traumatized controls, and that this impaired fear inhibition may be associated with altered HPA-axis functioning in PTSD (Jovanovic et al., 2010).

Animal studies have shown that proper fear conditioning and extinction learning require synaptic plasticity, and thus impaired synaptic plasticity may underlie impaired fear and extinction processes in PTSD (Mahan and Ressler, 2012). The BDNF-TrkB signaling pathway, a ligand-receptor system involved in synaptic plasticity, has been shown to be necessary for sustaining normal functioning of fear conditioning, extinction, and inhibitory learning in three brain regions, the amygdala, hippocampus, and medial PFC, all of which are associated with PTSD (Mahan and Ressler, 2012). Consolidation of fear conditioning and extinction was impaired when BDNF signaling was inhibited in the amygdala (Rattiner et al., 2004; Chhatwal et al., 2006). Heterogeneous *BDNF* knockout mice (*BDNF*±) demonstrated malfunctioning contextual fear conditioning, which can be partially reversed with recombinant BDNF infusion into the hippocampus (Liu et al., 2004). Altered BDNF expression in the prelimbic and infralimbic areas of the medial PFC can also lead to functional changes in fear consolidation and expression, suggesting a role of BDNF as a key mediator of neural plasticity in these regions (Choi et al., 2010; Peters et al., 2010). Glutamatergic and GABAergic signaling pathways have also been implicated in the regulation of fear consolidation, expression and extinction (Mahan and Ressler, 2012). For instance, disrupting NMDA and AMPA receptor functioning impaired the extinction of fear conditioning (Dalton et al., 2008; Liu et al., 2009; Zimmerman and Maren, 2010). Other ligand-receptor signaling systems such as those involving norepinephrine, nitric oxide, endocannabinoids, dopamine and acetylcholine have also been shown to play a modulatory role in the consolidation and extinction of fear conditioning, primarily by modulating glutamatergic and GABAergic signaling (Mahan and Ressler, 2012). These neurochemical systems involved in the fear circuitry provide potential pharmacological targets for reducing dysregulated fear response in PTSD and enhancing resilience to inappropriate fear associations in individuals susceptible to stress-related psychiatric disorders.

### Additional neural circuitry of resilience

Neural circuits underlying psychological characteristics that render adaptive social behavior and promote resilience in individuals have been examined. Psychobiological qualities important in prosocial behavior include emotion regulation, empathy, and altruism, among others (Charney, 2004; Feder et al., 2009). Animal and human studies have identified functional neural circuits and interactions among multiple brain regions, such as the amygdala, PFC and nucleus accumbens, that are involved in the regulation of adaptive psychobiological responses to stress and adversities (Charney, 2004; Feder et al., 2009; Kim et al., 2011b; Cusi et al., 2012; Morishima et al., 2012). Reduced Insular activation under stress has been linked to greater non-reactivity to inner experience, a key component of trait mindfulness which may protect against negative bias and reduce depression vulnerability (Paul et al., 2013). By potentially targeting the top-down and bottom-up regulation of these neural circuits, psychotherapeutic interventions including cognitive behavioral therapy with cognitive reappraisal, positive emotion exercises, coping skill training, well-being therapy, and mindfulness meditation, can be efficacious approaches to build and enhance resilient psychosocial responses to stress (Southwick and Charney, 2012).

## Summary

Resilience is a complex multidimensional construct and the study of its neurobiology is a relatively young area of scientific investigation (Southwick and Charney, 2012). Multiple interacting factors including genetics, epigenetics, developmental environment, psychosocial factors, neurochemicals, and functional neural circuitry, play critical roles in developing and modulating resilience in an integrated way. For instance, genetic and epigenetic factors interact with each other and determine the biological characteristics and regulation of neurochemicals and receptors. Environmental factors influence these characteristics and regulation processes through gene and environment interactions throughout development, contributing to adaptive changes in gene regulation, plasticity in the growth and modulation of neurocircuits, and the shaping of psychological factors and behavioral endpoints that underlie the manifestation of resilience.

Our growing understanding of the neurobiology of resilience has significant implications for the prevention and treatment of stress-related psychiatric disorders. Pharmacological interventions targeting the neurochemical systems involving NPY, BDNF, CRH, and HPA axis, among others, are being investigated as potential treatments for depression and PTSD. For instance, pharmacological agents targeting the hyperactivity and malfunction of HPA axis and CRH can possibly reduce the likelihood of pathological response to stress. Also, for individuals with altered NPYergic system, enhancing NPY levels and function may help to improve stress and anxiety regulation and to minimize the anxiogenic effects of CRH (Southwick and Charney, 2012).

Behavioral training targeting psychosocial risk factors and related neural pathways is also likely to increase resilience to stress (Karatsoreos and McEwen, 2011). Practice and training on enhancing stress-protective factors can lead to augmented plasticity and regulation of neural circuits that modulate reward and motivation, fear response, learning memory, emotion regulation, attention, cognitive executive function, adaptive social behavior, and cognitive reappraisal, thereby result in improved adaptation to stress and trauma, increased speed of recovery from adversities, and decreased susceptibility to stress-related psychopathology throughout life (Southwick and Charney, 2012). Furthermore, maintaining a supportive environment and providing resilience-building classes for child rearing can be particularly beneficial, in that children can learn how to master life challenges and acquire “stress inoculation” while growing up, enabling them to adaptively react to and master future challenges and stressors, thereby reducing susceptibility to stress-related psychopathology.

How to apply what we currently know about resilience to further the promotion of resilience and the prevention and treatment of stress-related psychopathology is one of the most critical questions for future studies. In addition, multidisciplinary research on the neurobiology of resilience should help to further identify risk and protective factors as well as their complex interactions and thereby facilitate the development of evidence-based interventions for enhancing resilience and mitigating risk for stress-related psychiatric disorders.

### Conflict of interest statement

The authors declare that the research was conducted in the absence of any commercial or financial relationships that could be construed as a potential conflict of interest.
